# Diagnostic accuracy of acute diverticulitis with unenhanced low‐dose CT

**DOI:** 10.1002/bjs5.50290

**Published:** 2020-05-20

**Authors:** A. Thorisson, M. Nikberg, M. R. Torkzad, H. Laurell, K. Smedh, A. Chabok

**Affiliations:** ^1^ Department of Radiology Västerås Sweden; ^2^ Centre for Clinical Research Uppsala University Västerås Sweden; ^3^ Colorectal Unit, Department of Surgery Region Västmanland Hospital Västerås Sweden; ^4^ Colorectal Unit, Department of Surgery Landstinget Dalarna Mora Sweden; ^5^ Department of Diagnostic Radiology The Royal Marsden NHS Foundation Trust Sutton UK

## Abstract

**Background:**

The aim of this study was to evaluate the diagnostic accuracy of unenhanced low‐dose CT (LDCT) in acute colonic diverticulitis in comparison with contrast‐enhanced standard‐dose CT (SDCT).

**Methods:**

All patients with clinically suspected diverticulitis who underwent LDCT followed by SDCT between January and October 2017 were evaluated prospectively. CT examinations were assessed for signs of diverticulitis, complications and other differential diagnoses by three independent radiologists (two consultants and one fourth‐year resident) using SDCT as the reference method. Sensitivity and specificity were calculated and Cohen's κ coefficient was used for agreement analyses.

**Results:**

Of the 149 patients included (mean age 66·7 years, M : F ratio 0·4), 107 (71·8 per cent) had imaging consistent with diverticulitis on standard CT. Sensitivity and specificity values for a diverticulitis diagnosis using LDCT were 95–99 and 86–100 per cent respectively, and respective values for identification of complications were 58–73 and 78–100 per cent. The corresponding κ values among the three readers for diagnosis were 0·984, 0·934 and 0·816, whereas κ values for complications were 0·680, 0·703 and 0·354. Of the 26 patients who presented with other causes of abdominal symptoms identified on standard CT, 23 were diagnosed correctly on LDCT. Missed cases included splenic infarction (1) and segmental colitis (2).

**Conclusion:**

The diagnostic accuracy of LDCT was high for the presence of acute diverticulitis. However, as signs of complicated disease can be missed using the low‐dose protocol, use of LDCT as a primary examination method should not preclude SDCT when complications may be suspected.

## Graphical abstract

Unenhanced low‐dose CT (LDCT) is as accurate as contrast‐enhanced standard‐dose CT (SDCT) for detection of acute colonic diverticulitis. However, smaller complications, such as pericolic abscesses and small amounts of extraluminal air, can be missed.

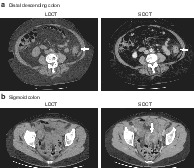

CT scan and diverticulitis

## Abstract (es)

**Antecedentes:**

Evaluar la precisión diagnóstica de la tomografía computarizada de dosis baja (*low‐dose computed tomography*, LDCT) sin contraste frente a la TC con dosis estándar (*standard‐dose* CT, SDCT) con contraste en la diverticulitis aguda de colon.

**Métodos:**

Todos los pacientes con sospecha clínica de diverticulitis aguda de colon a los que se realizó una LDCT seguida de una SDCT entre enero y octubre de 2017 se evaluaron prospectivamente. Tres radiólogos independientes (dos consultores y un residente de cuarto año) analizaron los signos de diverticulitis, complicaciones y otros diagnósticos diferenciales, utilizando la SDCT como método de referencia. Se calculó la sensibilidad y la especificidad, utilizándose el coeficiente κ de Cohen para los análisis de concordancia entre observadores.

**Resultados:**

De los 149 pacientes incluidos en el estudio (edad media 66,7 años, varón/mujer 0,4), 107 (71,8%) presentaban unas imágenes compatibles con diverticulitis en la SDCT. La sensibilidad y la especificidad para el diagnóstico de diverticulitis con la LDCT variaban entre el 95‐99% y el 86‐100%, respectivamente. La sensibilidad y la especificidad para la identificación de complicaciones oscilaron entre el 58‐73% y el 78‐100%, respectivamente. Los valores κ entre observadores para el diagnóstico fueron del 0,98, 0,93 y 0,82, respectivamente, mientras que para las complicaciones fueron del 0,68, 0,70 y 0,35. De los 26 pacientes en los que la SDCT identificó otras etiologías como causa de sus síntomas abdominales, 23 fueron diagnosticados correctamente con la LDCT. Los casos con diagnóstico erróneo correspondían a un infarto esplénico y dos colitis segmentarias.

**Conclusión:**

La precisión diagnóstica de la LDCT fue alta para detectar una diverticulitis aguda. Sin embargo, el protocolo de dosis baja puede no identificar los signos de la enfermedad complicada. Por lo tanto, su utilización como método de exploración primario no debe excluir la SDCT cuando se sospechen complicaciones.

## Introduction

The incidence of diverticulitis has increased during recent decades, and is reported to be up to 188 per 100 000 population per annum^1^, ^2^. The clinical presentation of diverticulitis is often non‐specific, and previous studies^3^, ^4^, ^5^ have shown that clinical judgement for suspected acute diverticulitis has an accuracy of about 50 per cent. Thus, radiological imaging is often required either to confirm or to exclude the diagnosis.

Several types of radiological examination can be performed on patients with suspected diverticulitis^6^. Ultrasonography can be used as the first‐line examination; although readily available and inexpensive, this method is time‐consuming and highly operator‐dependent^7^, ^8^, ^9^. MRI has been shown to be useful in diagnosing the condition, but is both expensive and time‐consuming^10^, ^11^, and may not be available in the acute setting. CT has high diagnostic accuracy, and is used as the primary examination method in many countries^5^.

Some authors recommend use of contrast‐enhanced standard‐dose CT (SDCT) with rectal contrast medium for the evaluation of suspected diverticulitis^12^. Recent studies^13^, ^14^ have used contrast‐enhanced SDCT but without oral or rectal contrast, and there is no consensus regarding CT examination modalities.

Exposure to ionizing radiation is the most concerning factor with CT examinations, and must be assessed and minimized as much as reasonably possible, as patients with diverticulitis often have recurrent inflammation, which requires repeated diagnostic studies^15^, ^16^. The aim of the present study was to evaluate whether unenhanced low‐dose CT (LDCT) is as accurate as SDCT in detecting suspected acute colonic diverticulitis.

## Methods

This prospective observational study was conducted in two hospitals located in Västerås and Mora, Sweden, and serving an area of 340 000 inhabitants. All consecutive patients admitted to the emergency department between January and October 2017 with clinically suspected diverticulitis were screened for enrolment. Patients received oral and written information about the radiation risks, and written informed consent was required for participation. The study was approved by the regional ethics committee and followed the 2013 Declaration of Helsinki guidelines (registration number 2016/411; clinical trials registration number NTC03443011).

### Study design

All patients aged 50 years and over presenting at the emergency room with clinically suspected diverticulitis, defined as pain in the lower left abdomen on physical examination, C‐reactive protein (CRP) level above 25 mg/l or white blood cell count greater than 10 × 10^9^/l, were asked to participate. Exclusion criteria included pregnancy, contraindication for contrast medium use (such as renal failure or allergy) and lack of informed consent. Clinical and demographic data were recorded.

### CT examination

Participants were examined first by LDCT followed directly by SDCT. All CT examinations were performed using a 64‐slice General Electric (GE) Optima CT660 machine (GE Healthcare, Marlborough, Massachusetts, USA). The CT examination protocols are shown in *Table* 1. All patients received iodine‐based intravenous contrast material Omnipaque™ (GE Healthcare) at a concentration of 350 mg iodine per ml over a constant injection time of 30 s. The contrast dosage was individualized based on the patient's age, height and weight using OmniVis™ software version 5.0 (GE Healthcare) for calculations; thus, the injection rate varied with the total contrast dosage. Time delay was individualized using SmartPrep (GE Healthcare) with a region of interest in the abdominal aorta (threshold 100 Hounsfield units) and calculated time delay for the portal venous phase. From the raw data for both CT acquisitions, 5‐mm slices in three planes were reformatted using iterative reconstructions, adaptive statistical iterative reconstructions and/or dose reduction. Patients received intravenous contrast only; no oral or rectal contrast was used. All contrast CT studies were done with the portal venous phase only.

**Table 1:** CT protocols

	Protocol	Approximate dose equivalence for 70‐kg person (mSv)
**Centre 1**		
LDCT	Full helical rotation: 0·6 s	3·5
	Dose: 120 kV	
	Noise index: 70	
	mA range: 50–400	
	Pitch: 1375 : 1	
	Iterative reconstructions with 50% ASIR but no dose reduction	
SDCT	Full helical rotation: 0·8 s	8·5
	Dose: 120 kV	
	Noise index: 36	
	mA range: 150–560	
	Pitch: 0984 : 1	
	Iterative reconstructions with 30% ASIR and 30% dose reduction	
**Centre 2**		
LDCT	Full helical rotation: 0·6 s	2·5–3·5
	Dose: 100 kV	
	Noise index: 60	
	mA range: 50–480	
	Pitch: 1375 : 1	
	Iterative reconstructions with 40% ASIR and 30% dose reduction	
SDCT	Full helical rotation: 0·6 s	10–12·5
	Dose: 120 kV	
	Noise index: 32	
	mA range: 120–560	
	Pitch: 0984 : 1	
	Iterative reconstructions with 30% ASIR and 30% dose reduction	

LDCT, unenhanced low‐dose CT; SDCT, contrast‐enhanced standard CT; ASIR, adaptive statistical iterative reconstruction.

### CT evaluation

CT evaluation was performed using a Sectra RIS and PACS system (IDS7 RIS and PACS version 19.1; Sectra Imtec, Linköping, Sweden). CT evaluation was performed separately by first reviewing all LDCT examinations, which were assessed for signs of diverticulitis, complications and other acute diagnoses. All readers were free to adjust window settings as preferred.

At a minimum of 4 weeks later, all full‐dose examinations were assessed using the same evaluation protocol. All examinations were evaluated by three independent radiologists: two senior abdominal radiology consultants with 5 and 15 years of experience respectively, and one fourth‐year radiology resident. All readers were blinded to patient outcome, previous CT findings, and one another's findings. Diagnosis of diverticulitis and occurrence of complication were based on consensus between the specialists on SDCT examinations. In case of disagreement between consultants about the presence of complications, CT scans were evaluated a second time together and a consensus was reached. SDCT was considered as the reference method in this study.

Diverticulitis was defined as colonic wall thickening greater than 5 mm, visible diverticula and pericolic fat stranding; these criteria applied to both LDCT and SDCT. Diverticular abscesses were defined as intramural, pericolic and pelvic collections. Signs of perforation were the occurrence of extraluminal air that was pericolic, retroperitoneal or in the peritoneal cavity. In doubtful cases, where the abscess was near the colonic wall, a cut‐off size of more than 15 mm was chosen to distinguish an inflamed diverticulum from a suspected abscess. This cut‐off was chosen because the risk of worsening outcomes for patients with a small abscess is low^17^, ^18^, ^19^.

For patients with an abscess on CT re‐evaluation, medical records and radiology reports were reviewed to determine whether the presence of abscess had been reported and for information on abscess drainage.

### Statistical analysis

SDCT was used as the reference method and has been shown^20^, ^21^ to have 94–95 per cent sensitivity and 96–99 per cent specificity for diverticulitis.

A two‐sided *P* value below 0·050 was considered significant. The power calculation was based on the confidence interval (c.i.) for sensitivity without consideration for specificity. An anticipated sensitivity of 0·95 for LDCT and a half c.i. width of 0·03 indicated a need for a total of 104 patients with diverticulitis. A presumed clinical accuracy rate of 65 per cent and a drop‐out rate of 10 per cent gave an anticipated total number of participants of 176. However, admission protocols for participants noted confirmation of diverticulitis on inclusion CT and were reviewed regularly by a research nurse. When a minimum of 104 patients with SDCT‐confirmed diverticulitis had been included, further inclusion in the study was stopped.

Sensitivity was calculated for LDCT using consensus between specialists on SDCT as the reference method.

Intraobserver and interobserver agreements were assessed using the κ value and respective asymptotic standard error (ASE). Agreement was considered poor for κ below 0·2, low for κ of 0·21–0·40, moderate for κ of 0·41–0·60, good for κ of 0·61–0·80, and excellent for κ above 0·80^22^. All data analysis was performed using IBM SPSS® version 24.0 (IBM, Armonk, New York, USA).

## Results

Of 272 patients screened for enrolment during the study period, 149 were included in the study. Their mean(s.d.) age was 66·7(10·7), and 107 were women.

Three patients aged less than 50 years (ages 39, 40 and 42 years) were enrolled as they met the clinical criteria and agreed to participate with informed consent. The mean(s.d.) BMI was 28·8(4·9) (range 19·5–51·4) kg/m^2^. Mean(s.d.) body temperature was 37·4(0·7)°C, CRP level 82(61) mg/l, and white blood cell count 11·8(4·4) × 10^9^/l.

**Figure 1:**
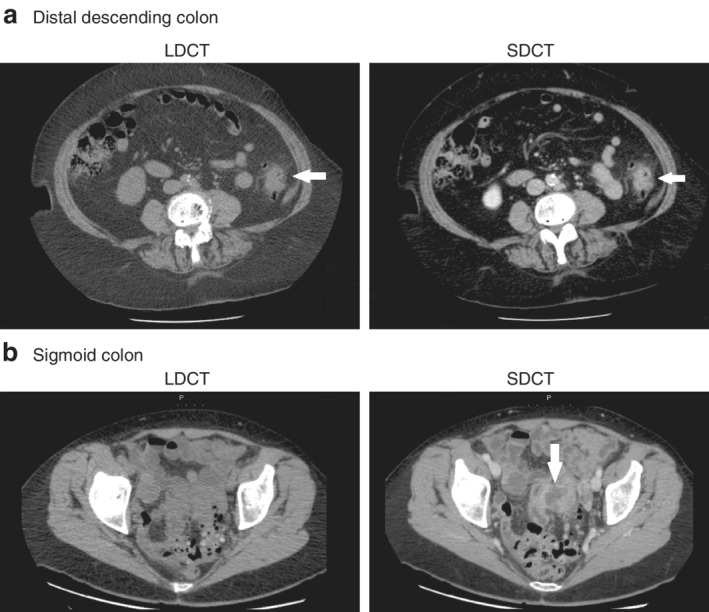
Diverticulitis diagnosed using the two CT protocols **a** Diverticulitis in the distal descending colon (arrows) shown with unenhanced low‐dose CT (LDCT) and contrast‐enhanced standard‐dose CT (SDCT) protocols. **b** Axial images of diverticulitis in the sigmoid colon of a different patient. Diverticula, colonic wall thickening and pericolic fat stranding are seen with both CT examination types. However, an abscess between the sigmoid colon and an adjacent small bowel loop can be detected only with the SDCT protocol (arrow).

Some 107 (71·8 per cent) of the 149 included patients met the criteria for diverticulitis according to the reference method (SDCT) and consensus between consultants was reached; these patients were therefore considered to have true cases of acute colonic diverticulitis (*Fig*. 1). The sigmoid colon was the site of inflammation in 77 patients (72·0 per cent) and the descending colon for the remaining 30 (28·0 per cent).

**Figure 2:**
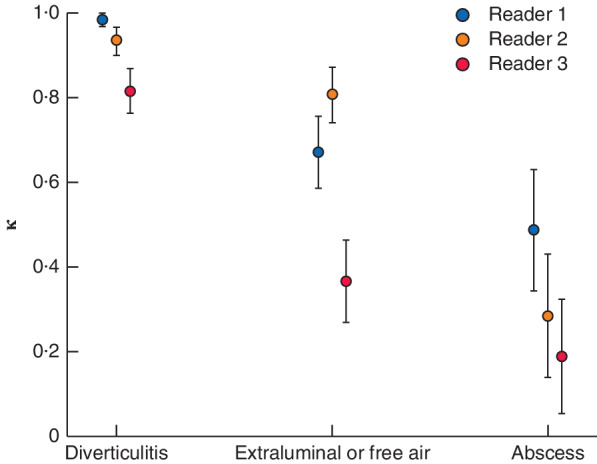
Weighted κ values for unenhanced low‐dose CT for the presence of diverticulitis, extraluminal air and abscesses for the three readers For diagnosis of diverticulitis all 149 patients were included, but for other variables only the 107 patients with diverticulitis were included. κ values and their respective asymptotic standard errors are shown, using consensus on contrast‐enhanced standard‐dose CT as the reference for each reader. Readers 1 and 2 were consultant radiologists; reader 3 was a fourth‐year resident radiologist.

The overall sensitivity of LDCT for diagnosing acute diverticulitis was 98·6 per cent (211 of 214) for the two consultants combined, and the overall specificity was 98 per cent (82 of 84) (*Table* 2). Intraobserver agreement for the presence of diverticulitis on LDCT was excellent for the consultants and good for the resident (*Fig*. 2). The sensitivity, specificity and agreement for LDCT compared with the reference standard for diverticulitis using consensus on SDCT were: 99·1 per cent, 100 per cent and κ = 0·984(ASE 0·016) respectively for reader 1; 98·1 per cent, 95 per cent and κ = 0·934(0·033) for reader 2; and 95·3 per cent, 86 per cent and κ = 0·816(0·053) for the radiology resident (*Table* 2).

**Table 2:** Frequency of findings on unenhanced low‐dose CT for sensitivity and specificity for all readers

		Consultant reader 1			Consultant reader 2			Resident reader 3		
	Consensus	LDCT	Sensitivity	Specificity	LDCT	Sensitivity	Specificity	LDCT	Sensitivity	Specificity
Diverticulitis	107	106	106 of 107 (99)	42 of 42 (100)	105	105 of 107 (98)	40 of 42 (95)	102	102 of 107 (95)	36 of 42 (86)
Extraluminal or free air	28	22	17 of 28 (61)	78 of 79 (99)	24	24 of 28 (86)	74 of 79 (94)	34	11 of 28 (39)	62 of 79 (78)
Abscess	13	5	5 of 13 (38)	93 of 94 (99)	3	3 of 13 (23)	92 of 94 (98)	2	2 of 13 (15)	92 of 94 (98)

Values in parentheses are percentages. All calculations are intraobserver calculations with consensus between specialists on standard‐dose CT as reference method. The presence of diverticulitis was calculated for all 149 included patients; extraluminal/free air and abscess calculations were done only for the 107 patients with diverticulitis. A few patients had both extraluminal/free air and abscess assessed by all readers. LDCT, unenhanced low‐dose CT.

Reasons for the presentation of abdominal symptoms other than diverticulitis were found in 26 patients on SDCT, of which 23 (88 per cent) were diagnosed correctly by at least two readers using the LDCT protocol. One case of splenic infarction and two cases of segmental colitis were missed in the LDCT group. The most common differential diagnoses for the patient group were colitis (8 patients) and appendicitis (7). In total, 16 patients had either a negative finding on CT or no consensus on diagnosis; no definitive cause for these patients' symptoms could be found with either scanning method.

### Complications

Overall, signs of complicated diverticulitis were found in 26, 39 and 41 patients for the three readers using SDCT. For 33 patients there was a consensus on complicated diverticulitis. The sensitivity of LDCT with regard to the presence of any complication of diverticulitis was 61 per cent (20 of 33) and 73 per cent (24 of 33) for the consultants, and 58 per cent (19 of 33) for the radiology resident (*Table* 2). The respective κ values were 0·680(0·079), 0·703(0·076) and 0·354(0·096). The specificity of LDCT for complications was 100 per cent (74 of 74) and 95 per cent (70 of 74) for the two consultants, and 78 per cent (58 of 74) for the resident.

The median maximum diameter of abscesses was 2·2 (range 1·6–3·7) cm. The sensitivity for the presence of an abscess was 38 per cent (5 of 13), 23 per cent (3 of 13) and 15 per cent (2 of 13) when consensus for LDCT and SDCT were compared for readers 1, 2 and 3 respectively (*Table* 2). Corresponding κ values were 0·487(0·143), 0·285(0·145) and 0·189(0·135).

The sensitivity of LDCT for extraluminal or free air in patients with diverticulitis was 61 per cent (17 of 28) and 86 per cent (24 of 28) for the consultants, and 39 per cent (11 of 28) for the resident. Corresponding κ values were 0·672(0·085), 0·807(0·065) and 0·367(0·097). Specificity values for complications are shown in *Table* 2.

Of the 13 patients with an abscess, as agreed by consensus, six had an abscess recorded in the initial CT report, and small amounts of extraluminal air were reported for three of these patients. Four patients were reported as having uncomplicated diverticulitis. One patient was readmitted after 2 weeks because of deteriorating clinical status, and repeat CT showed progress of the abscess that required CT‐guided drainage. No patient required emergency surgical intervention, and there was no mortality.

### Interobserver agreement

**Figure 3:**
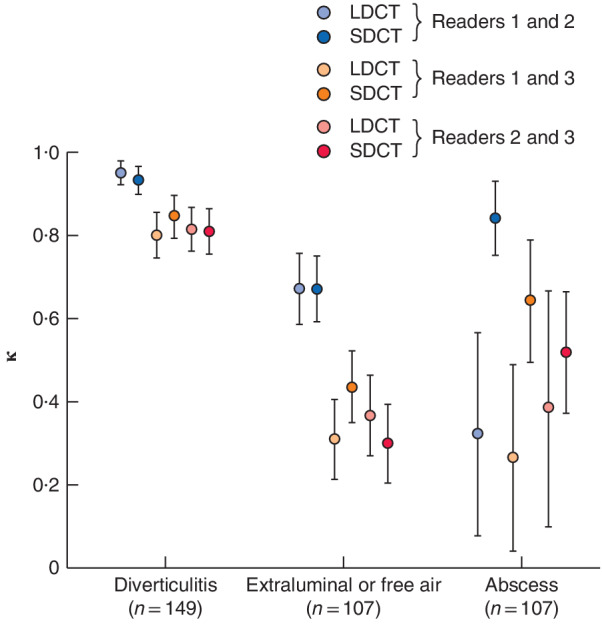
Comparison of interobserver agreement on the two CT protocols for the presence of diverticulitis, extraluminal air and abscesses for the three readers κ values and their respective asymptotic standard errors are shown. LDCT, unenhanced low‐dose CT; SDCT, contrast‐enhanced standard‐dose CT. Readers 1 and 2 were consultant radiologists; reader 3 was a fourth‐year resident radiologist.

Interobserver agreement was evaluated using the respective readers' SDCT as the reference method (*Table* 3). Interobserver agreement was excellent for the presence of diverticulitis for the two consultants, and good between the consultants and the resident. Agreement for extraluminal or free air was good between the two consultants regardless of the imaging method, and fair between the consultants and the resident (*Fig*. 3).

**Table 3:** Frequency of findings for readers on low‐dose and standard‐dose CT

	Consultant reader 1				Consultant reader 2				Resident reader 3			
	LDCT	SDCT	Sensitivity	Specificity	LDCT	SDCT	Sensitivity	Specificity	LDCT	SDCT	Sensitivity	Specificity
Diverticulitis	106	107	106 of 107 (99·1)	43 of 43 (100)	107	108	107 of 108 (99·1)	41 of 41 (100)	102	111	102 of 111 (91·9)	32 of 38 (84)
Extraluminal or free air	18	24	18 of 24 (75)	83 of 83 (100)	28	34	25 of 34 (74)	70 of 73 (96)	34	40	21 of 40 (53)	54 of 67 (81)
Abscess	5	9	5 of 9 (56)	98 of 98 (100)	3	12	2 of 12 (17)	94 of 95 (99)	2	6	2 of 6 (33)	101 of 101 (100)

Values in parentheses are percentages. The presence of diverticulitis was calculated for all 149 included patients; extraluminal/free air and abscess calculations were done only for the 107 patients with diverticulitis. LDCT, unenhanced low‐dose CT; SDCT, contrast‐enhanced standard‐dose CT.

Interobserver agreement was low for the presence of abscesses between the two consultants on LDCT, but excellent on SDCT. Agreement on abscesses was either low or moderate between the two consultants and the resident, and the agreement improved between one consultant and the resident on SDCT (*Fig*. 3).

### Radiation doses

As with all CT examinations, radiation doses vary according to patient body size, because larger patients require higher radiation doses to achieve the same image quality^23^. In this study, the protocols resulted in a mean(s.d.) radiation dose of about 3·3(1·9) mSv for LDCT and about 10·9(4·8) mSv for SDCT. Thus, the mean(s.d.) radiation dose for LDCT was about 30(6) per cent that for SDCT. All radiation doses were estimated from each patient's respective dose–length product for the separate CT protocols. The mean(s.d.) CT dose index was 4·32(2·36) mGy for LDCT and 14·75(5·60) mGy for SDCT.

## Discussion

This study suggests that, although LDCT may be sensitive for the detection of diverticulitis with excellent intermodality agreement, it has significantly lower sensitivity for the detection of complications. LDCT could be the method of choice in patients with a mild clinical status and previous history of diverticulitis, to minimize exposure to ionizing radiation and the potential risk of nephrotoxicity and allergic reactions in patients with recurrent diverticulitis.

However, the present findings suggested that LDCT should not be the first‐line radiological examination, as patients with minor complications often have a mild clinical status and could therefore be missed using a LDCT protocol. SDCT was shown to have greater sensitivity and specificity for the presence of a diverticular abscess.

The intermodality agreement for the presence of complications was equal to or higher than the interobserver agreement. This suggests that detection of subtle complications is often more dependent on the reader than the CT protocol.

The abscesses in question were small (median diameter just over 2 cm), and some were not reported initially. Patients with a small abscess missed on the initial report did not return to hospital and were managed successfully without antibiotics on an outpatient basis. However, a negative finding on LDCT for complications in a patient with deteriorating or severe clinical status should warrant additional examination with SDCT, even though such patients are rare according to the results of this and other studies^17^, ^24^.

In a previous study^25^, the LDCT protocol had similar accuracy to SDCT in patients with suspected acute diverticulitis, and was documented to be sensitive for the detection of both diverticulitis and diverticular abscess, although abscess size was not reported.

Variation in abdominal CT experience between readers was estimated to evaluate whether LDCT was sufficient for all levels of examiner, not just for abdominal subspecialist radiologists. As expected, some variation in agreement was reported, but seemed to be dependent more closely on the reader than on the CT method, as shown by the higher κ values for intermodality compared with interobserver variation.

The diagnosis was missed in three patients using LDCT, and was made in these patients only on SDCT. Therefore, in patients with a negative LDCT and severe clinical status, further investigation with SDCT may be appropriate.

A possible limitation of this study is a risk of recall bias; this was mitigated partially by reviewing SDCT examinations after 4 weeks. Another limitation was the use of SDCT as the standard, although this is the method of choice in current clinical practice.

Previous studies^3^, ^4^, ^5^ have shown the accuracy of clinical suspicion for diverticulitis to be about 50 per cent, whereas in these series 72 per cent of the included patients had diverticulitis. This may represent a selection bias, with included patients having a high suspicion of diverticulitis.

Although use of LDCT reduces radiation exposure in patients with suspected diverticulitis, this examination has lower sensitivity for complications and cannot be recommended as the first‐line radiological investigation for patients with clinically suspected acute complicated diverticulitis.
